# Predictors of placebo response in pharmacological and dietary supplement treatment trials in pediatric autism spectrum disorder: a meta-analysis

**DOI:** 10.1038/tp.2015.143

**Published:** 2015-09-22

**Authors:** A Masi, A Lampit, N Glozier, I B Hickie, A J Guastella

**Affiliations:** 1 Autism Clinic for Translational Research, Brain and Mind Centre, Central Clinical School, Faculty of Medicine, University of Sydney, Camperdown, NSW, Australia; 2 Regenerative Neuroscience Group, Brain and Mind Centre, Central Clinical School, Faculty of Medicine, University of Sydney, Camperdown, NSW, Australia

## Abstract

Large placebo responses in many clinical trials limit our capacity to identify effective therapeutics. Although it is often assumed that core behaviors in children with autism spectrum disorders (ASDs) rarely remit spontaneously, there has been limited investigation of the size of the placebo response in relevant clinical trials. These trials also rely on caregiver and clinical observer reports as outcome measures. The objectives of this meta-analysis are to identify the pooled placebo response and the predictors of placebo response in pharmacological and dietary supplement treatment trials for participants with a diagnosis of ASD. Randomized controlled trials (RCTs) in pediatric ASD, conducted between 1980 and August 2014, were identified through a search of Medline, EMBASE, Web of Science, Cochrane Database of Systematic Reviews and clinicaltrials.gov. RCTs of at least 14 days duration, comparing the treatment response for an oral active agent and placebo using at least one of the common outcome measures, were included. Analysis of 25 data sets (1315 participants) revealed a moderate effect size for overall placebo response (Hedges' *g*=0.45, 95% confidence interval (0.34–0.56), *P*<0.001). Five factors were associated with an increase in response to placebo, namely: an increased response to the active intervention; outcome ratings by clinicians (as compared with caregivers); trials of pharmacological and adjunctive interventions; and trials located in Iran. There is a clear need for the identification of objective measures of change in clinical trials for ASD, such as evaluation of biological activity or markers, and for consideration of how best to deal with placebo response effects in trial design and analyses.

## Introduction

Autism spectrum disorder (ASD) is characterized by core deficits in social communication and interaction, and the presence of restricted, repetitive patterns of behavior, interests or activities. Prevalence is estimated to be as high as 1 in 68.^1^ There are currently no medication treatments approved for the core symptoms of ASD. The US Food and Drug Administration has approved two atypical antipsychotics for children with ASD, risperidone and aripiprazole, for irritability symptoms, including tantrums, aggression and self-injury behaviors. Established pharmacological and dietary supplement interventions have also been trialed as treatments for behavioral symptoms, such as repetitive behavior, aggression, hyperactivity and irritability,^2, 3, 4, 5^ and for social impairment.^6^ However, little evidence exists to support the efficacy for most of these treatments.^7, 8^ Notwithstanding the lack of established evidence for medications, it has been estimated that at least 25% of children with ASD take at least one medication.^9^

The assessment of treatment response within clinical trials presents a major challenge to establishing efficacious interventions to treat core symptoms of ASD and associated social and behavioral impairments. Inconsistent results from clinical trials for core social and communication impairment in ASD have been a noted feature that has limited drug development.^10^ Although it is often assumed that core behaviors are unlikely to remit spontaneously in children with severe ASD, randomized controlled trials (RCTs) have found that up to 30% of child ASD participants respond to placebo treatments.^11^ Outcome measures for children with ASD continue to be dependent on observer and informant ratings.^12, 13^ Although it is widely recommended that both independent and caregiver ratings are employed to reduce rating biases, it is unclear how observer and informant ratings are independently influenced by placebo effects and impact on the evaluation of treatment outcome of trials in ASD. In a double-blinded, placebo-controlled randomized trial treating children with autistic disorder, results for parent-rated outcome measures were nonsignificant, whereas statistically significant improvements were reported for clinician-rated scales.^11^ Conversely, in an RCT assessing the effects of oxytocin on social behaviors in adolescents with ASD, parental beliefs moderated outcome, regardless of whether the child was actually assigned the active drug condition.^6^

Previous reviews in psychiatric patient populations have identified trial design factors and patient characteristics that moderate the placebo response within clinical trials. In schizophrenia, shorter duration of illness, greater baseline symptom severity, younger age and trials of shorter duration were associated with greater placebo response,^14^ and more recent trials were associated with a greater placebo effect.^15^ Antidepressant trials in adults showed response to placebo for outcomes rated by observers was significantly greatly than outcomes completed by patients,^16^ and in pediatric trials higher baseline severity was associated with lower placebo response.^17^ Similarly, in a secondary analysis of baseline factors in a multi-site RCT in ASD, lower symptom severity at baseline predicted increased response to placebo.^18^

There are few treatment options in ASD. It is imperative that design of, and recruitment for, RCTs is well informed. To our knowledge, there has been no systematic or meta-analytic review evaluating the placebo response and its moderators in ASD to aid identification of strategies to control these factors. Specifically, whether the type of observer rating the outcome measure used to report treatment response has an impact on response to placebo needs to be clarified. The aims of this study are to undertake a systematic review and meta-analysis of RCTs of pharmacological and dietary supplement treatments for symptoms associated with the core deficits and associated symptoms in children with ASD in order to evaluate the placebo response effect size and determine patient and trial characteristics that may predict placebo response. Given prior considerations of how placebos can influence caregiver perceptions and behaviors, we hypothesized that the mean change pre-post treatment with placebo would be greater for parent or caregiver raters compared with clinician raters.

## Materials and methods

The systematic review and meta-analysis were undertaken and reported in accordance with the PRISMA statement (Preferred Reporting Items for Systematic Reviews and Meta-Analysis).^19^

### Data sources and study selection

**Figure 1:**
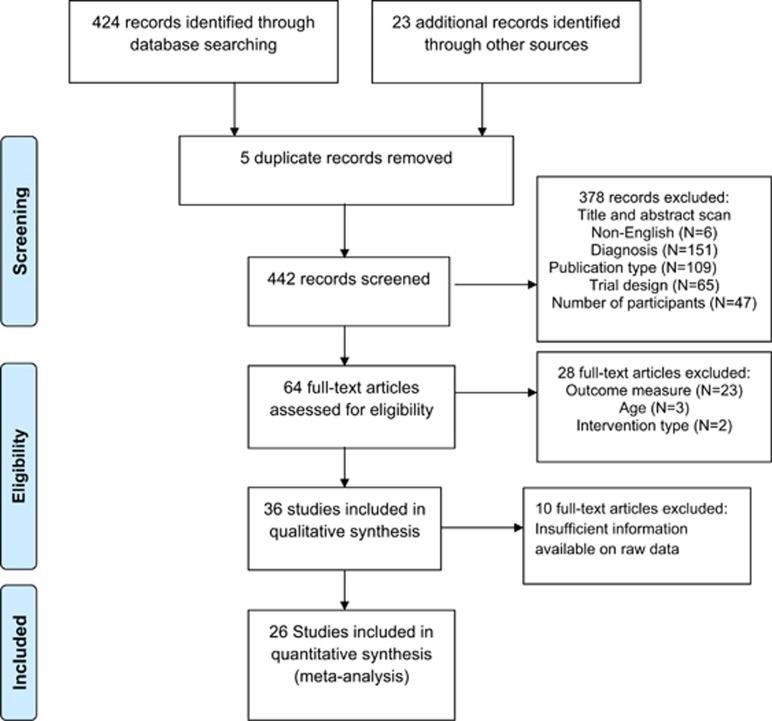
Flow of information through the stages of the meta-analysis, including reasons for excluding a full-text article.

MEDLINE, EMBASE, Web of Science, Cochrane Database of Systemic Reviews and clinicaltrials.gov databases were searched for articles published in English from January 1980 to August 31 2014 using the keywords ((‘autism' or ‘asperger' or ‘pervasive developmental disorder') AND (‘placebo') AND (‘randomized')). After removal of duplicates, two reviewers (AM and Kerribeth Szolusha) independently screened search results based on title and abstract. The full text of remaining studies identified as meeting inclusion criteria, as described below, were then reviewed and agreement reached between researchers (Kerribeth Szolusha and AM) on eligibility of each study. The reference lists of eligible studies were searched for studies meeting inclusion criteria. A flowchart detailing the stages of the assessment of studies was constructed according to PRISMA guidelines^19^ (Figure 1).

Eligible studies included published, peer-reviewed articles reporting results from double-blind RCTs comparing treatment response between active agent and placebo using either parallel or crossover designs with at least 10 participants per arm, for at least 14 days. Participants were aged 3 to 20 years and diagnosed with autistic disorder, Asperger's syndrome or pervasive developmental disorder according to the Diagnostic and Statistical Manual of Mental Disorders III or IV, Autism Diagnostic Interview-Revised or Autism Diagnostic Observation Schedule. Interventions were pharmacological or dietary supplement treatments, taken orally and compared against placebo. Studies reported means and s.d. for each group at baseline and end point (defined as when the intervention was last given). A recent review identified the most commonly used outcome measures assessing treatment response in ASD,^20^ highlighting 289 unique measurement tools used to record response/outcome. Only three tools were used more than 5% of the time across all studies to measure cognitive/behavioral symptoms/skills: Aberrant Behavior Checklist (ABC), Clinical Global Impression rating scales (CGI) and the Vineland Adaptive Behavior Scales. The ABC, CGI and the Childhood Autism Rating Scale were the three most commonly used outcome measurement tools in pharmacological treatment trials. Therefore, trials using the ABC, CGI, Vineland Adaptive Behavior Scales and Childhood Autism Rating Scale as outcome measures to assess treatment response were included. In addition, the Children's Yale-Brown Obsessive-Compulsive Scale (CY-BOCS) and the CY-BOCS modified for Pervasive Developmental Disorders (CY-BOCS-PDD) were included as a measure of change in repetitive behavior.^21^ Both primary and secondary outcomes were considered.

### Data extraction

Baseline and endpoint data for outcome measures assessing treatment response in active intervention and placebo groups were extracted into an excel spreadsheet (mean, s.d. and sample size for each group and time point). Two reviewers (AM and Kerribeth Szolusha) independently extracted all the data and ensured accuracy. In crossover design trials only data for the first phase were extracted. When continuous data were reported in formats other than means and s.d., we contacted the authors to request raw data.

In order to explore potential moderators of placebo response, we also extracted the following descriptive variables: type of rater, type of outcome measure (primary or secondary), type of active intervention, adjunctive treatment status (defined as a combination of pharmacological agent approved for use in ASD with another pharmacological agent or dietary supplement not approved for treatment in ASD), baseline severity (reported as ABC-Irritability subscale), number of contact visits for assessment purposes, trial duration, length of washout period, number of study sites, study location, study quality, publication year, mean age, gender (% males) and sample size. The type of rater was categorized as clinician (which included trained raters and evaluators), caregiver (which included parents) or clinician including caregiver interview. If it was not stated, and authors had not responded to a request for information, it was assumed that the parent or caregiver of the participant completed the ABC as is usual.^22^

The Jadad scale^23^ and the Cochrane Collaboration's tool for assessing risk of bias^24^ were used to evaluate methodological quality of each RCT included in the meta-analysis. Jadad is an 11-item instrument, with three items directly related to the control of bias and eight items related to study design and features. The maximum possible score is 13. The Cochrane Collaboration's risk of bias tool requires an assessment of the risk of bias associated with specific features as ‘low risk', ‘high risk' or ‘unclear risk'.

### Data analysis

All analyses were performed using Comprehensive Meta-Analysis Version 2 (Biostat, Englewood, NJ, USA). The primary outcome was standardized mean difference (SMD, calculated as Hedges' *g*) from baseline to end point between groups (active treatment and placebo, using a pre-post correlation of 0.7) as well as within the placebo group. SMDs of 0.2, 0.5 and 0.8 were considered small, moderate and large, respectively.^25^ If a study reported baseline and endpoint data for multiple subscales, such as the subscales in the ABC,^22^ a single effect estimate per study was calculated based on mean SMD and variance across outcomes.

Analysis of placebo response across studies was conducted by pooling combined SMDs using a random-effects model. In order to explore potential moderators of placebo response, we performed subgroup meta-analyses using a mixed-effects model. A mixed-effects model uses a random-effects model to combine outcomes within subgroups and a fixed-effects model to compare subgroups.^26^ In addition, we performed univariate meta-regressions to investigate the possible impact of continuous moderators on placebo effect size across studies. Cochrane's *Q*-statistic was used to test between-subgroup heterogeneity.^24^

Forest plots were used to identify outliers and potential sources of heterogeneity. The impact of any identified outlier was assessed by removing the study reporting the outlier and comparing the subsequent effect size and *P*-value to the initial result. The *I*^2^-statistic was used to assess true heterogeneity across studies (that is, the proportion of heterogeneity across studies that is not due to random error), with values of 25, 50 and 75% implying small, moderate and high levels of heterogeneity, respectively.^27^ Small study effect resulting from publication bias, insufficient reporting of outcomes, selective inclusion of study participants or other sources was assessed by visually inspecting funnel plots of SMDs against s.e.^28^ and tested using Egger's test of the intercepts.^29^ A trim and fill analysis for random-effects models was used to estimate the impact of small study effect on pooled estimates.^30^

## Results

A total of 26 studies were assessed as eligible for quantitative analysis after 447 studies were identified in the initial search (Figure 1). One study^31^ reported on secondary outcome measures not included in the initial study, resulting in 25 unique data sets. The data set comprised 1315 participants (*N* active treatment=661, *N* placebo=654). Male participants comprised 80% of the sample size, and age ranged from 3 to 18 years, with the exception of one study^3^ where the upper end of the age range was 20 years. Table 1 presents study characteristics from the placebo-controlled trials. Nine studies reported the target of the active intervention as behavior,^32, 33, 34, 35, 36, 37, 38, 39, 40^ whereas seven studies reported the target of intervention as irritability.^5, 41, 42, 43, 44, 45^ The remaining studies reported the target of intervention as hyperactivity,^4, 46^ repetitive behavior,^2, 13^ disruptive symptoms,^47, 48^ autism severity,^49^ aggression^3^ and the core symptoms of autism.^50, 51^ There were 107 data points measuring effect size, representing an estimate of the magnitude of the difference in outcome measure between baseline and end point, for active intervention and placebo treatment groups.

Using the Jadad scale, study quality scores varied between 8 and 13 with an average score of 11.6 (s.d.=1.8). In all, 11 out of 25 studies scored a maximum of 13 points. Assessment of quality using the Cochrane Risk of Bias Tool found 18 out of 25 studies were judged to be ‘low risk' overall. Supplementary Table S1 presents the risk assessment for each study across every domain. The proportion of studies within each risk level is shown in Supplementary Figure S1.

### Overall effect size of placebo response

**Figure 2:**
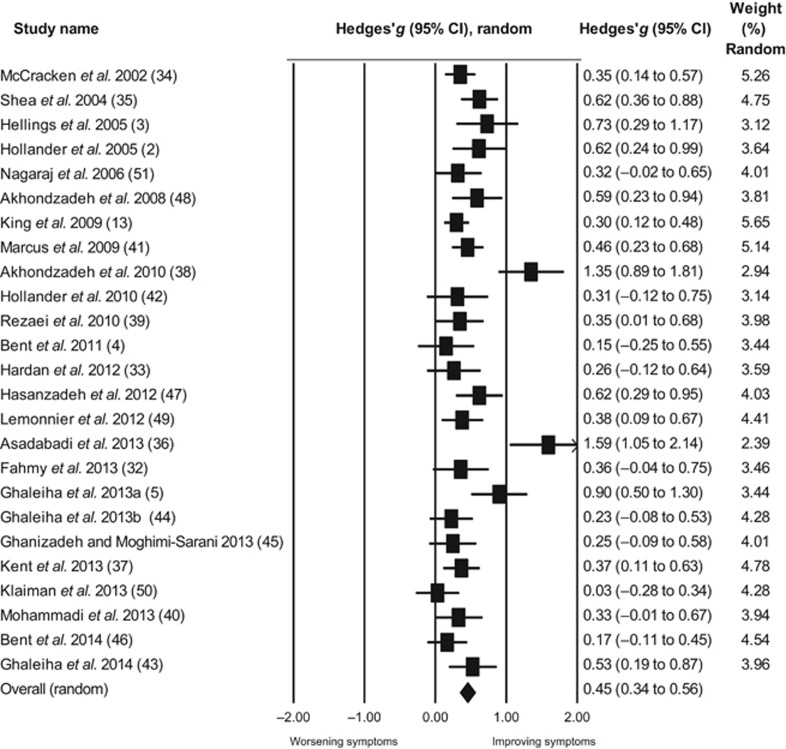
Forest plot of efficacy of treatment with placebo. The arrow representing the study of Asadabadi *et al.* 2013 (ref. 36) shows the confidence interval spread further than the limit for the effect size range (−2.00 to 2.00). CI, confidence interval.

The size of the placebo response across studies was moderate and statistically significant (*k*=25, *g*=0.45, 95% confidence interval (CI) (0.34–0.56), *P*<0.001, Figure 2). The level of true heterogeneity was moderate (*I*^2^=62.89%), meaning that only about 40% of the heterogeneity was due to random error. The funnel plot showed significant asymmetry (Egger's intercept=2.85, *P*=0.02; Supplementary Figure S2). In a trim and fill analysis, the adjusted effect size after imputation of one study was *g*=0.42 (95% CI (0.31–0.54)). In a subsequent sensitivity analysis, two notable outlier studies were then removed from the analysis.^36, 38^ The overall effect size of placebo remained statistically significant (*k*=23, *g*=0.39, 95% CI (0.31–0.46), *P*<0.001). The resulting heterogeneity across the remaining studies was small (*I*^2^=26.47%) and the funnel plot did not show significant asymmetry (Egger's intercept=1.01, *P*=0.33; Supplementary Figure S3). Nonetheless, these two studies were included in all subsequent analyses.

### Moderators of placebo response

**Figure 3:**
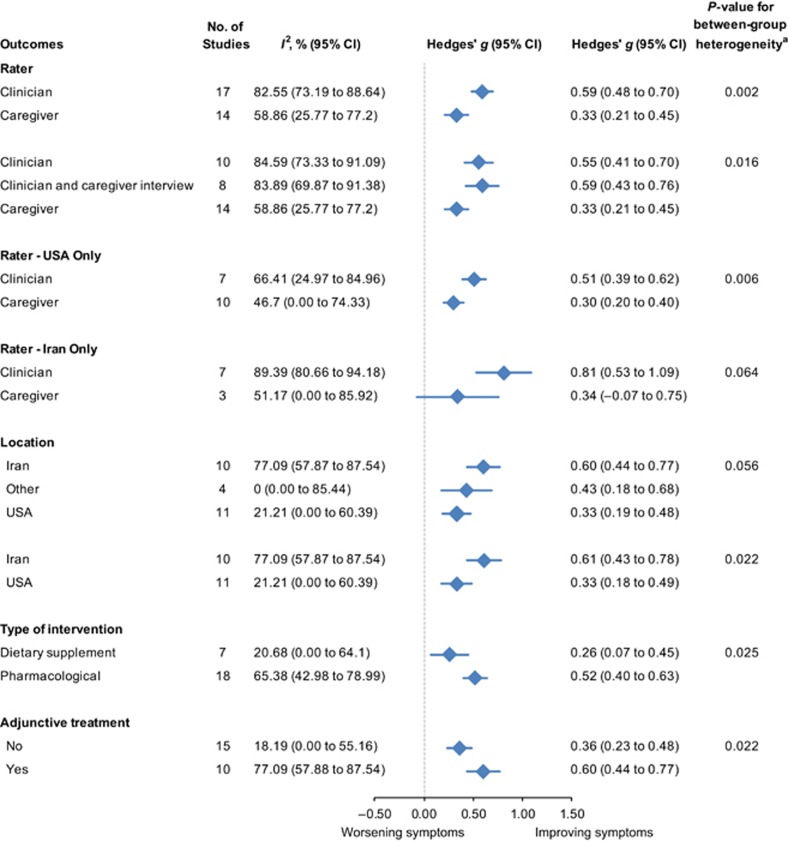
Subgroup analyses of moderators of placebo response in randomized controlled trials in pediatric ASD. ^a^*Q*-test for between-group heterogeneity, mixed-effects model. ASD, autism spectrum disorder; CI, confidence interval.

Potential moderators of placebo response were then investigated in subgroup analyses (Figure 3). Initially, in order to address the proposed hypothesis that the response to placebo would be greater for caregiver ratings compared with clinician ratings, raters were classified based on who completed the outcome measure. For ratings categorized as clinician, the ratings were based on either direct observation by the clinician or on information obtained during an interview with the caregiver conducted by the clinician, or a combination of both methods. One study^46^ included an outcome measure rated by teachers, which was excluded from this analysis on the basis that teachers may not have been specifically trained to assess change. There was a significantly higher placebo response for outcome measures rated by a clinician compared with measures rated by a caregiver (*Q*-statistic for between-subgroup heterogeneity=9.72, df=1, *P*=0.002; Figure 3). The effect of rater was further explored by separating the assessments completed by a clinician with input from a caregiver through an interview, from those completed exclusively by a clinician or a caregiver. The significant effect for rater remained, with moderate effect size estimates for both the clinician-rated group and the group that was rated by a clinician, which included input from a caregiver interview. Effect size estimates for ratings completed by a caregiver remained small (*Q*=8.28, df=2, *P*=0.02; Figure 3).

In addition, the effect of rater was investigated for Iran and the United States, the two countries where the majority of trials were conducted. A significant difference in efficacy based on clinician ratings remained for trials located in the United States compared with measures rated by the caregiver (*Q*=7.44, df=1, *P*=0.006; Figure 3). There was a trend toward a significant effect for clinician ratings for trials located in Iran compared with measures rated by a caregiver (*Q*=3.44, df=1, *P*=0.06; Figure 3).

Trials of pharmacological interventions had a moderate placebo effect size significantly greater than the small placebo effect size seen in trials of dietary supplements (*Q*=5.02, df=1, *P*=0.03; Figure 3). Trials of adjunctive interventions, which included both pharmacological and dietary supplement treatments, also had a moderate placebo effect size significantly greater than the small placebo effect size in monotherapy trials (*Q*=5.26, df=1, *P*=0.02; Figure 3). There was no difference in the placebo effect size for primary outcome measures compared with secondary outcome measures (*Q*=3.20, df=1, *P*=0.07).

We then examined the influence of trial location. A trend in the magnitude of placebo response was shown. Trials located in Iran reported a moderate placebo effect size compared with a small effect size for trials located in other countries and the United Sates (*Q*=5.64, df=2, *P*=0.06; Figure 3). The placebo response was significantly greater in Iran than the United States (*Q*=5.27, df=1, *P*=0.02; Figure 3).

The impact of continuous modifiers on placebo response rates across studies was then investigated using random-effects meta-regression analyses. The year of publication (*β*=−0.01, *P*=0.49), the number of participants in the placebo group (*β*=0.00, *P*=0.49), number of contact visits (*β*=0.01, *P*=0.52), the age of participants (*β*=0.01, *P*=0.15), trial duration (*β=*-0.002, *P*=0.24), the severity of presentation at start of trial (*β*=0.00, *P*=0.84) and the study quality assessed using the Jadad scale (*β*=−0.03, *P*=0.32) did not demonstrate any significant influence on the placebo response.

**Figure 4:**
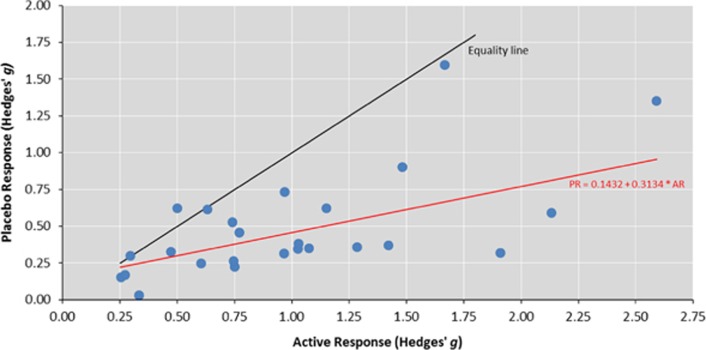
Placebo response versus response to treatment with active intervention. The equality line has a slope equal to 1, where the response to placebo is the same as the response to active intervention. The point above the line represents a trial where response to placebo was greater than response to treatment with the active intervention. A larger vertical divergence from the equality line represents a larger response to active intervention compared with response to placebo. The line labeled PR=0.1432+0.3134 × AR represents the regression line. AR, active response; PR, placebo response.

To facilitate the examination of whether response to active intervention is a predictor of the level of response to placebo, a further meta-analysis of the efficacy of active intervention was undertaken. This resulted in a large and statistically significant effect size for the overall treatment response to the active intervention (*k*=25, *g*=0.96, 95% CI (0.79–1.14), *P*<0.001; Supplementary Figure S4). Heterogeneity across studies was large (*I*^2^=96.45%). Meta-regression revealed the magnitude of placebo response was significantly influenced by the response to active intervention (*β*=0.31, *P*<0.001, Figure 4). The proportion of between-study variation in effect size explained by the response to active intervention was 49%. The ratio of the overall effect sizes for the active and placebo treatment groups implies that 47% of improvements in the active treatment group were attributable to the placebo effect.

## Discussion

This meta-analysis has demonstrated that the response to treatment with placebo across the 25 trials in pediatric ASD is moderate. This challenges assumptions that have often been made about the lack of change in core behavioral features in children with ASD over relatively short periods of time. Five factors predicted a greater placebo response: the rater of the outcome measure, the response to active intervention, the type of active intervention, an adjunctive treatment and the geographical location of the trial. Contrary to the hypothesis, ratings by clinicians were associated with a stronger placebo effect. Similarly, a stronger response to the active intervention, the use of a pharmacological or an adjunctive treatment in a trial, and studies conducted in Iran, also predicted greater placebo response.

The role of the observer and rater of outcome measures used in RCTs in pediatric ASD is crucial, as evidenced by the inability to identify any self-report outcome measures in all the qualifying studies of this meta-analysis. The significant effect of ratings by clinicians on placebo response may reflect rater bias driven by underlying beliefs, motivations and enthusiasm for a potential efficacious treatment.^16, 52, 53^ The potential for overestimation of positive effects by clinicians would be supported by our results. Alternatively, caregiver burden may diminish placebo effects and thereby increase the differential between ratings by clinicians and caregivers. Previously, increased caregiver strain has been found to be associated with lower placebo response, potentially reflecting reduced hopefulness or optimism at the time of the child's entry into the study.^18^

Response to active intervention was strongly associated with the response to placebo. This interesting association has implications for sample size and design of phase-2 and -3 trials testing interventions that have had phase-1 trial success. Larger studies will be required to detect genuine differences between active medications and placebos, if medications are indicating initial effectiveness from phase-1 study. In addition, this result suggests that factors predicting the degree of placebo response in the trial may be a major driver of the effect size within the active arm. Placebo response is estimated to contribute up to 50% of the response to pain medication, and up to 75% of the positive effects in trials for antidepressant medication.^54^ In treatment trials for patients with unipolar depressive disorder, improvements in placebo groups corresponded to 67% of the improvement in the active treatment groups.^16^ Using a similar methodology for the trials included in this meta-analysis, 47% of the improvement in the active treatment group could be attributable to the placebo effect. Factors that may have led to an improvement in both treatment and placebo arms include overall quality of care (although we note US trials had the lowest improvement), multiple contact visits with clinicians and raters, the amount of time spent with participants or the amount of additional support given to participants.^54^ Alternatively, rater knowledge or expectation about the potential benefits of an active intervention may have influenced the level of response in both groups. Further research investigating factors that are differentially associated with response to active intervention and placebo is required.

Rater expectation may have led to greater improvement in placebo response in RCTs of pharmacological interventions than for trials of dietary supplement treatments. This finding is consistent with previous analyses of RCTs in major depressive disorder, where it was proposed that participants in complementary and alternative medicine trials, which included dietary supplements, may have more modest expectations than participants in trials of pharmacological interventions.^55^ The use of risperidone, one of only two pharmacological interventions approved for treatment of children with ASD, as the adjunctive treatment in all included trials, may have influenced rater expectations and led to a greater improvement in RCTs of adjunctive treatments. Unlike previous placebo meta-analyses in mood disorders,^14, 15^ there appeared no temporal trend in the placebo response in RCTs for ASD. In addition, baseline severity was also not associated with greater or reduced response to placebo, in contrast to the single, but multi-site RCT study by King *et al.*^18^

This study has also shown that the level of response to placebo varied depending on the geographical location of the RCT, with response being greater for trials conducted in Iran compared with the United States. Differences in participant recruitment procedures and characteristics, and cultural dynamics may have led to systematically greater placebo response in studies conducted in Iran.^56^ Alternatively, there may be differences in age of diagnosis and access to early intervention services across countries that may alter the susceptibility to the effects of expectancy on response to treatment with placebo. This requires further investigation.

The results of this study highlight the urgent need to develop valid and objective measures of baseline assessment and treatment response in ASD. Inclusions based on objective measures, which are differentiated from the primary outcome variable, are yet to be fully explored and identified. However, there is preliminary evidence that suggests certain neuropsychological, physiological or other neurobiological markers, driven by an understanding of underlying biochemical, physiological and structural changes in ASD, should be considered for screening, and baseline and endpoint measures of response to intervention as a means to reduce placebo response.^57, 58, 59^

The number of RCTs meta-analyzed was limited by the small size of a number of trials that did not meet inclusion criteria, which subsequently prevented exploration of the interdependence of potential contributors to placebo response. Specifically, we note that all trials of adjunctive treatments were located in Iran, and therefore the effect of geography on placebo response in trials of adjunctive treatments could not be investigated. In RCTs conducted in pediatric major depression, the strongest predictor of placebo response was the number of study sites.^17^ Only 4 out of 25 studies included in this meta-analysis were multi-site trials, so the effect of the number of study sites could not be adequately investigated. Few trials included a placebo washout-screening phase, which prevented an investigation of whether blinded randomization to an initial phase of placebo treatment reduces the observed response to placebo.

## Conclusions

This meta-analysis has demonstrated a moderate placebo effect in RCTs of pediatric ASD, and identified five key factors that increase the placebo effect size: outcome measures completed by clinicians, the level of response to active intervention, a pharmacological active intervention, adjunctive treatments and the geographical location of the trial. The impact of these factors should be considered in the trial design phase in order to minimize the placebo effect, improve the detection of active treatment and placebo differences and subsequently improve identification of efficacious treatments for the symptoms of ASD. Although it is generally accepted that both caregiver and clinician ratings should be used as baseline and endpoint measures, findings also highlight the need for objective measures, such as evaluation of biological activity or markers, to ensure comprehensive screening and assessment of response to treatment.

## Figures and Tables

**Table 1:** Study characteristics

*Study demographics—Placebo*					*Study design*							*Study quality*
*Study*	N	*Age (mean)*	*% Male*	*Location*	*Active treatment*	*Adjunctive treatment*	*Type of active treatment*	*Treatment duration (days)*	*Number of study sites*	*Outcome measure*	*Rater*	*Jadad score*
Akhondzadeh *et al.*^48^	20	6.8	70	Iran	Piracetam	Yes (risperidone)	Pharmacological	70	1	P: ABC (total score)	Clinician and caregiver interview	13
Akhondzadeh *et al.*^38^	20	7.4	70	Iran	Pentoxifylline	Yes (risperidone)	Pharmacological	70	1	P: ABC-I S: ABC-H, IS, L, SB	Clinician and caregiver interview	13
Asadabadi *et al.*^36^	20	7.5	65	Iran	Celecoxib	Yes (risperidone)	Pharmacological	70	1	P: ABC-I S: ABC-H, IS, L, SB	Clinician	13
Bent *et al.*^4^	13	5.8	84.6	USA	Omega-3 fatty acid	No	Dietary supplement	84	1	P: ABC-H S: ABC-I, IS, L, SB	Caregiver	12
Bent *et al.*^46^	28	7.1	85.7	USA	Omega-3 fatty acid	No	Dietary supplement	42		P: ABC-H S: ABC-I, IS, L, SB	Caregiver	13
Fahmy *et al.*^32^	14	5.7	78.6	Egypt	L-carnitine	No	Dietary supplement	168	1	S: CARS	Clinician	11
Ghaleiha *et al.*^5^	20	8	60	Iran	Memantine	Yes (risperidone)	Pharmacological	70	1	P: ABC-I S: ABC-H, IS, L, SB	Clinician and caregiver interview	12
Ghaleiha *et al.*^44^	24	7.6	66.7	Iran	Riluzole	Yes (risperidone)	Pharmacological	70	1	P: ABC-I S: ABC-H, IS, L, SB	Caregiver	13
Ghaleiha *et al.*^43^	23	5.9	78.3	Iran	Galantamine	Yes (risperidone)	Pharmacological	70	1	P: ABC-I S: ABC-H, IS, L, SB	Caregiver	13
Ghanizadeh and Moghimi-Sarani^45^	20	7.9	60	Iran	*N*-acetylcysteine	Yes (risperidone)	Dietary supplement	56	1	P: ABC-I S: ABC-H, IS, L, SB	Caregiver	12
Hardan *et al.*^33^	15	7.2	100	USA	*N*-acetylcysteine	No	Dietary supplement	84	1	P: ABC-I S: ABC-H, IS, L, SB; CGI-S	Clinician; caregiver	11
Hasanzadeh *et al.*^47^	24	6.8	83.3	Iran	*Ginkgo biloba*	Yes (risperidone)	Dietary supplement	70	1	P: ABC-I S: ABC-H, IS, L, SB	Clinician and caregiver interview	13
Hellings *et al.*^3^	14	10.3	100	USA	Valproate	No	Pharmacological	23	1	ABC-I; CGI-S; CGI-I	Clinician; caregiver	13
Hollander *et al.*^2^	20	7.4	85	USA	Fluoxetine	No	Pharmacological	42	1	P: CY-BOCS; S: CGI-AD	Clinician	8
Hollander *et al.*^42^	11	9	90.9	USA	Divaloprex sodium	No	Pharmacological	84	1	S: ABC-I	Caregiver	10
Kent *et al.*^37^	35	9	88.6	USA	Risperidone	No	Pharmacological	42	16	P: ABC-I S: CGI-S	Clinician; caregiver	12
King *et al.*^13^	76	9.6	84.2	USA	Citalopram	No	Pharmacological	84	6	P: CY-BOCS-PDD S: ABC-I, H, IS, L, SB	Clinician; caregiver	12
Klaiman *et al.*^50^	23	5	78.3	USA	Tetrahydra-biopterin	No	Dietary supplement	112	1	S: ABC-I, H, IS, L, SB	Caregiver	13
Lemonnier *et al.*^49^	30	7.1	73.3	France	Bumetanide	No	Pharmacological	84	1	P: CARS	Clinician and caregiver interview	9
Marcus *et al.*^41^	52	10.2	92.3	USA	Aripiprazole	No	Pharmacological	56	37	S: ABC-H, IS, L, SB; CGI-S; CY-BOCS (compulsion subscale)	Clinician; caregiver	9
McCracken *et al.*^34^	52	8.8	82.7	USA	Risperidone	No	Pharmacological	56	6	P: ABC-I S: ABC-H, IS, L, SB; VABS; CY-BOCS	Clinician; clinician and caregiver interview; caregiver	8
Mohammadi *et al.*^40^	20	7.1	85	Iran	Amantadine	Yes (risperidone)	Pharmacological	70	1	P: ABC-I S: ABC-H, IS, L, SB	Clinician	13
Nagaraj *et al.*^51^	21	5.3	85.7	India	Risperidone	No	Pharmacological	168	1	P: CARS	Clinician and caregiver interview	12
Rezaei *et al.*^39^	20	7.9	70	Iran	Topiramate	Yes (risperidone)	Pharmacological	56	1	P: ABC-I S: ABC-H, IS, L, SB	Clinician and caregiver interview	13
Shea *et al.*^35^	39	7.3	82.1	Canada	Risperidone	No	Pharmacological	63	1	P: ABC-I S: ABC-H, IS, L, SB	Caregiver	8

Abbreviations: ABC, aberrant behavior checklist; ABC-H, ABC-hyperactivity/noncompliance subscale; ABC-I, ABC-irritability subscale; CARS, childhood autism rating scale; CGI, clinician global impression; CGI-I, CGI improvement; CGI-S, CGI severity; CY-BOCS, Children's Yale-Brown Obsessive-Compulsive Scale; CY-BOCS-PDD, CY-BOCS modified for pervasive developmental disorders; IS, ABC-inappropriate speech subscale; L, ABC-lethargy/social withdrawal subscale; P, primary outcome measure; S, secondary outcome measure; SB, ABC-stereotypic behavior subscale; VABS, Vineland Adaptive Behavior Scale.

## Supplementary Material

Supplementary Informations
