# Supplementary material for: Predictors of placebo response in pharmacological and dietary supplement treatment trials in pediatric autism spectrum disorder: a meta-analysis
Source: Transl Psychiatry. 2015 Sep 22;5(9):e640–. doi: 10.1038/tp.2015.143 (PMC5068810; doi:10.1038/tp.2015.143)
Supplement: Supplementary Informations [file tp2015143x1.pdf]

Table S1: Cochrane Risk of bias within studies

Study	Sequence generation	Allocation concealment	Participant blinding	Personnel blinding	Assessor blinding	Incomplete outcome data	Selective outcome reporting	Other sources of bias	Summary
Akhondzadeh 2008 (1)	Low	Low	Low	Low	Low	Low	Low	Low	Low
Akhondzadeh 2010 (2)	Low	Low	Low	Low	Low	Low	Low	Low	Low
Asadabadi 2013 (3)	Low	Low	Low	Low	Low	Low	Low	Low	Low
Bent 2011 (4)	Low	Low	Low	Low	Low	Low	Low	Low	Low
Bent 2014 (5)	Low	Low	Low	Low	Low	Low	Low	Low	Low
Fahmy 2013 (6)	Low	Low	Low	Low	Low	High	Low	Low	High
Ghaleiha 2013a (7)	Low	Low	Low	Low	Low	Low	Low	Low	Low
Ghaleiha 2013b (8)	Low	Low	Low	Low	Low	Low	Low	Low	Low
Ghaleiha 2014 (9)	Low	Low	Low	Low	Low	High	Low	Low	High
Ghanizadeh 2013 (10)	Low	Low	Low	Low	Low	High	Low	Low	High
Hardan 2012 (11)	Low	Low	Low	Low	Low	Low	Low	Low	Low
Hasanzadeh 2012 (12)	Low	Low	Low	Low	Low	Low	Low	Low	Low
Hellings 2005 (13)	Unclear	Low	Low	High	Low	Unclear	High	Low	High
Hollander 2005 (14)	Unclear	Unclear	Low	Low	Low	High	Low	Low	High
Hollander 2010 (15)	Unclear	Low	Low	Low	Low	Low	Low	Low	Low
Kent 2013 (16)	Low	Low	Low	Low	Low	High	Low	Low	High
King 2009 (17)	Low	Low	Low	Low	Low	Low	Low	Low	Low
Klaiman 2013 (18)	Low	Low	Low	Low	Low	Low	Low	Low	Low
Lemonnier 2012 (19)	Unclear	Low	Low	Low	Low	Low	Low	Low	Low
Marcus 2009 (20)	Unclear	Low	Low	Low	Low	High	Low	Low	High
McCracken 2002 (21)	Unclear	Low	Low	Unclear	Low	Low	Low	Low	Low
Mohammadi 2013 (22)	Low	Low	Low	Low	Low	Low	Low	Low	Low
Nagaraj 2006 (23)	Low	Low	Low	Low	Low	Low	Low	Low	Low
Rezaei 2010 (24)	Low	Low	Low	Low	Low	Low	Low	Low	Low
Shea 2004 (25)	Unclear	Low	Low	Low	Low	Low	Low	Low	Low

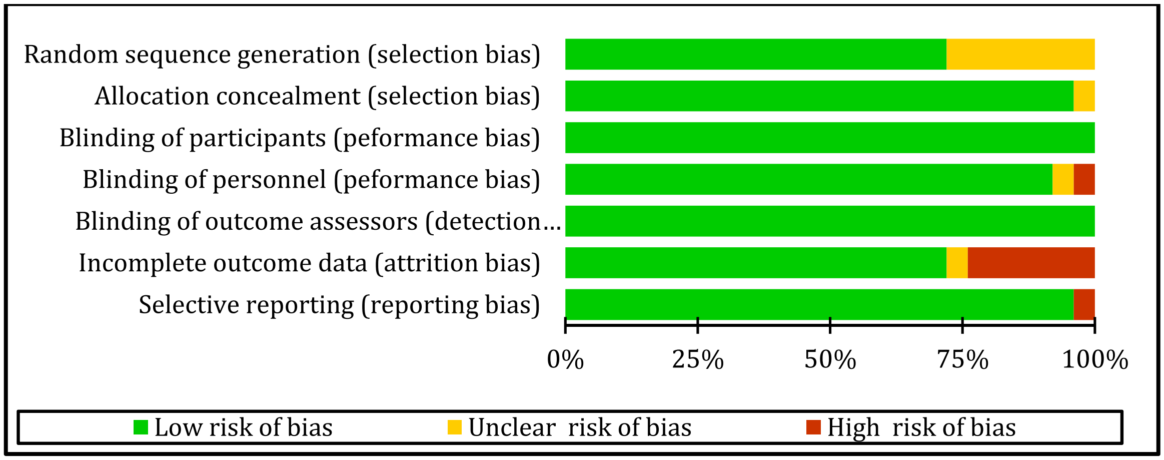

Figure S1: Cochrane risk of bias graph, illustrating the proportion of studies within each category of risk for each domain.

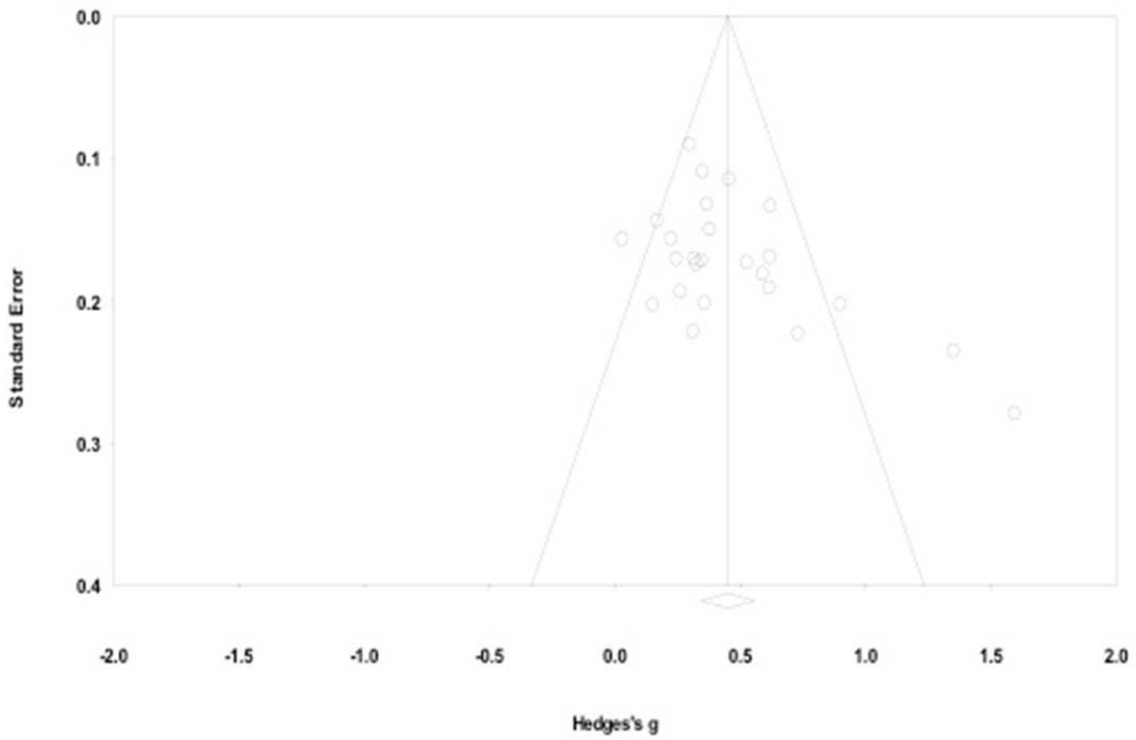

Figure S2: Funnel Plot for assessing publication bias: Overall placebo effect (N=25).

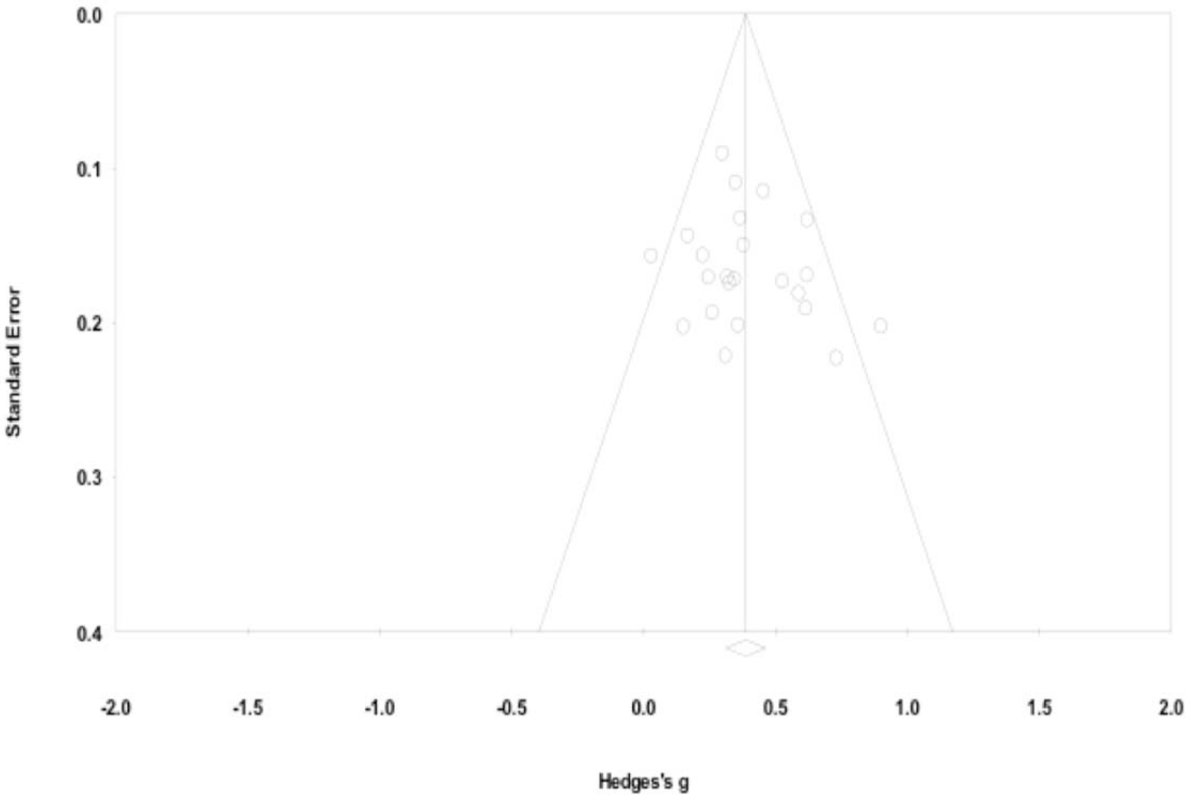

Figure S3: Funnel Plot for assessing publication bias: Overall placebo effect excluding two outlier studies (2,3) (N=23)

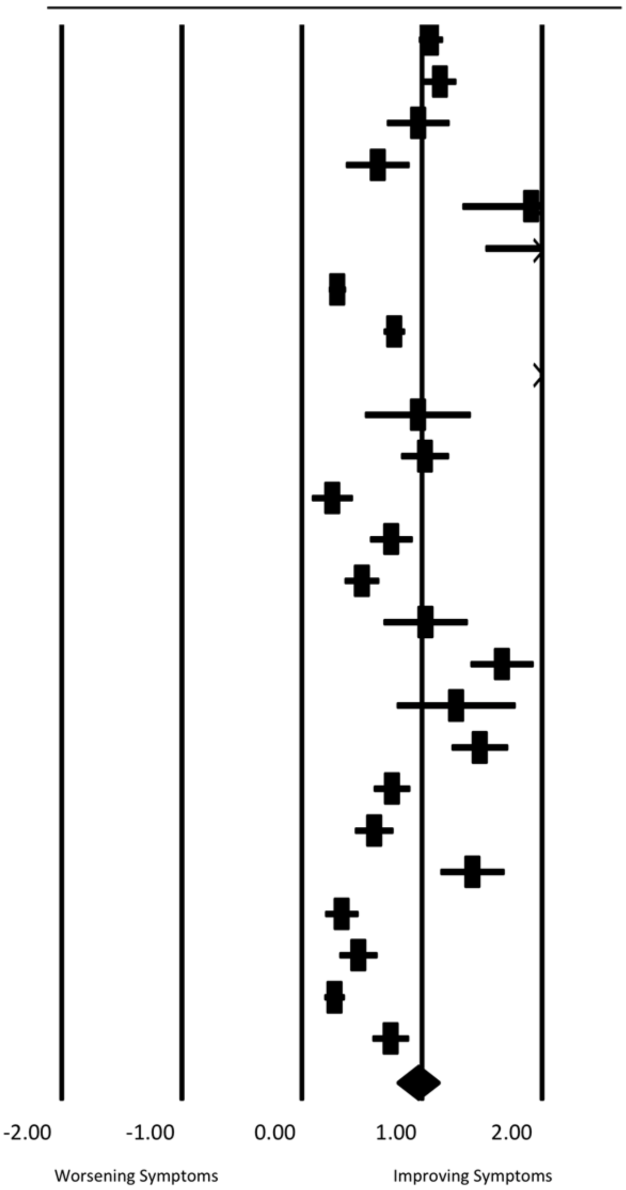

Study name	Weight (%) Random	Hedges' *g* (95% CI)
McCracken 2002 (21)	4.33	1.07 (0.97 to 1.18)
Shea 2004 (25)	4.28	1.15 (1.01 to 1.29)
Hellings 2005 (13)	4.00	0.97 (0.70 to 1.23)
Hollander 2005 (14)	3.99	0.63 (0.36 to 0.90)
Nagaraj 2006 (23)	2.99	1.91 (1.33 to 2.49)
Akhondzadeh 2008 (1)	2.89	2.13 (1.53 to 2.74)
King 2009 (17)	4.37	0.29 (0.22 to 0.37)
Marcus 2009 (20)	4.35	0.77 (0.68 to 0.86)
Akhondzadeh 2010 (2)	3.84	2.59 (2.27 to 2.91)
Hollander 2010 (15)	3.44	0.97 (0.52 to 1.41)
Rezaei 2010 (24)	4.16	1.03 (0.82 to 1.23)
Bent 2011 (4)	4.22	0.25 (0.08 to 0.43)
Hardan 2012 (11)	4.21	0.74 (0.56 to 0.93)
Hasanzadeh 2012 (12)	4.27	0.50 (0.35 to 0.65)
Lemonnier 2012 (19)	3.73	1.03 (0.68 to 1.38)
Asadabadi 2013 (3)	4.00	1.67 (1.40 to 1.93)
Fahmy 2013 (6)	3.25	1.29 (0.79 to 1.78)
Ghaleiha 2013a (7)	4.07	1.48 (1.24 to 1.72)
Ghaleiha 2013b (8)	4.26	0.75 (0.60 to 0.91)
Ghanizadeh 2013 (10)	4.24	0.60 (0.44 to 0.77)
Kent 2013 (16)	3.98	1.42 (1.15 to 1.69)
Klaiman 2013 (18)	4.28	0.33 (0.19 to 0.47)
Mohammadi 2013 (22)	4.24	0.47 (0.31 to 0.63)
Bent 2014 (5)	4.35	0.27 (0.18 to 0.36)
Ghaleiha 2014 (9)	4.25	0.74 (0.58 to 0.90)
Overall (random)		0.96 (0.79 to 1.14)

Figure S4: Forest plot for treatment response to active intervention (N = 25). CI: Confidence Interval

References

1. Akhondzadeh S, Tajdar H, Mohammadi MR, Mohammadi M, Nouroozinejad GH, Shabstari OL, *et al.* A double-blind placebo controlled trial of piracetam added to risperidone in patients with autistic disorder. *Child Psychiatry Hum Dev* 2008; 39:237-245.

2. Akhondzadeh S, Fallah J, Mohammadi MR, Imani R, Mohammadi M, Salehi B, *et al.* Double-blind placebo-controlled trial of pentoxifylline added to risperidone: Effects on aberrant behavior in children with autism. *Prog Neuropsychopharmacol Biol Psychiatry* 2010; 34:32-36.

3. Asadabadi M, Mohammadi MR, Ghanizadeh A, Modabbernia A, Ashrafi M, Hassanzadeh E, *et al.* Celecoxib as adjunctive treatment to risperidone in children with autistic disorder: A randomized, double-blind, placebo-controlled trial. *Psychopharmacology* 2013; 225:51-59.

4. Bent S, Bertoglio K, Ashwood P, Bostrom A, Hendren RL. A pilot randomized controlled trial of omega-3 fatty acids for autism spectrum disorder. *J Autism Dev Disord* 2011; 41:545-554.

5. Bent S, Hendren RL, Zandi T, Law K, Choi JE, Widjaja F, *et al.* Internet-based, randomized, controlled trial of omega-3 fatty acids for hyperactivity in autism. *J Am Acad Child Adolesc Psychiatry* 2014; 53:658-666.

6. Fahmy SF, El-Hamamsy MH, Zaki OK, Badary OAL-Carnitine supplementation improves the behavioral symptoms in autistic children. *Research in Autism Spectrum Disorders* 2013; 7:159-166.

7. Ghaleiha A, Asadabadi M, Mohammadi MR, Shahei M, Tabrizi M, Hajiaghaee R, *et al.* Memantine as adjunctive treatment to risperidone in children with autistic disorder: A randomized, double-blind, placebo-controlled trial. *Int J Neuropsychopharmacol* 2013a; 16:783-789.

8. Ghaleiha A, Mohammadi E, Mohammadi MR, Farokhnia M, Modabbernia A, Yekehtaz H, *et al.* Riluzole as an adjunctive therapy to risperidone for the treatment of irritability in children with autistic disorder: A double-blind, placebo-controlled, randomized trial. *Pediatric Drugs* 2013b; 15:505-514.

9. Ghaleiha A, Ghyasvand M, Mohammadi M-R, Farokhnia M, Yadegari N, Tabrizi M, *et al.* Galantamine efficacy and tolerability as an augmentative therapy in autistic children: A randomized, double-blind, placebo-controlled trial. *J Psychopharmacol (Oxf)* 2014; 28:677-685.

10. Ghanizadeh A, Moghimi-Sarani E. A randomized double blind placebo controlled clinical trial of N-Acetylcysteine added to risperidone for treating autistic disorders. *BMC Psychiatry* 2013; 13:196.

11. Hardan AY, Fung LK, Libove RA, Obukhanych TV, Nair S, Herzenberg LA, *et al.* A randomized controlled pilot trial of oral N-acetylcysteine in children with autism. *Biol Psychiatry* 2012; 71:956-961.

12. Hasanzadeh E, Mohammadi MR, Ghanizadeh A, Rezazadeh SA, Tabrizi M, Rezaei F, *et al.* A double-blind placebo controlled trial of Ginkgo biloba added to risperidone in patients with autistic disorders. *Child Psychiatry Hum Dev* 2012; 43:674-682.

13. Hellings JA, Weckbaugh M, Nickel EJ, Cain SE, Zarcone JR, Reese RM, *et al.* A double-blind, placebo-controlled study of valproate for aggression in youth with pervasive developmental disorders. *J Child Adolesc Psychopharmacol* 2005; 15:682-692.

14. Hollander E, Phillips A, Chaplin W, Zagursky K, Novotny S, Wasserman S, *et al.* A placebo controlled crossover trial of liquid fluoxetine on repetitive behaviors in childhood and adolescent autism. *Neuropsychopharmacology* 2005; 30:582-589.

15. Hollander E, Chaplin W, Soorya L, Wasserman S, Novotny S, Rusoff J, *et al.* Divalproex sodium vs placebo for the treatment of irritability in children and adolescents with autism spectrum disorders. *Neuropsychopharmacology* 2010; 35:990-998.

16. Kent J, Kushner S, Ning X, Karcher K, Ness S, Aman M, *et al.* Risperidone Dosing in Children and Adolescents with Autistic Disorder: A Double-Blind, Placebo-Controlled Study. *J Autism Dev Disord* 2013; 43:1773-1783.

17. King BH, Hollander E, Sikich L, McCracken JT, Scahill L, Bregman JD, *et al.* Lack of efficacy of citalopram in children with autism spectrum disorders and high levels of repetitive behavior: Citalopram ineffective in children with autism. *Arch Gen Psychiatry* 2009; 66:583-590

18. Klaiman C, Huffman L, Masaki L, Elliott GR. Tetrahydrobiopterin as a treatment for autism spectrum disorders: A double-blind, placebo-controlled trial. *J Child Adolesc Psychopharmacol* 2013; 23:320-328.

19. Lemonnier E, Degrez C, Phelep M, Tyzio R, Josse F, Grandgeorge M, *et al.* A randomised controlled trial of bumetanide in the treatment of autism in children. *Translational Psychiatry* 2012; 2:e202.

20. Marcus RN, Owen R, Kamen L, Manos G, McQuade RD, Carson WH, *et al.* A Placebo-Controlled, Fixed-Dose Study of Aripiprazole in Children and Adolescents With Irritability Associated With Autistic Disorder. *J Am Acad Child Adolesc Psychiatry* 2009; 48:1110-1119.

21. McCracken JT, McGough J, Shah B, Cronin P, Hong D, Aman MG, *et al.* Risperidone in children with autism and serious behavioral problems. *N Engl J Med* 2009; 347:314-321.

22. Mohammadi MR, Yadegari N, Hassanzadeh E, Farokhnia M, Yekehtaz H, Mirshafiee O, *et al.* Double-blind, placebo-controlled trial of risperidone plus amantadine in children with autism: A 10-week randomized study. *Clin Neuropharmacol* 2013; 36:179-184.

23. Nagaraj R, Singhi P, Malhi P. Risperidone in children with autism: Randomized, placebo-controlled, double-blind study. *J Child Neurol* 2006; 21:450-455.

24. Rezaei V, Mohammadi MR, Ghanizadeh A, Sahraian A, Tabrizi M, Rezazadeh SA, *et al.* Double-blind, placebo-controlled trial of risperidone plus topiramate in children with autistic disorder. *Prog Neuropsychopharmacol Biol Psychiatry* 2010; 34:1269-1272.

25. Shea S, Turgay A, Carroll A, Schulz M, Orlik H, Smith I, *et al.* Risperidone in the treatment of disruptive behavioral symptoms in children with autistic and Other pervasive developmental disorders. *Pediatrics* 2004; 114:e634-e641.
